# Treg in CNS autoimmune disease

**DOI:** 10.1186/1479-5876-10-S3-I14

**Published:** 2012-11-28

**Authors:** Stephen M Anderton

**Affiliations:** 1 MRC Centre for Inflammation Research, University of Edinburgh, UK

## 

The cellular interactions underlying the inflammatory component of MS are complex and involve counteracting pro-inflammatory and regulatory lymphocyte populations. Our understanding of these has been influenced by the use of increasingly sophisticated versions of experimental autoimmune encephalomyelitis (EAE) in the mouse. CD4+ T cells are the key drivers of EAE, although the exact means by which they exert their pathogenic influence are still not fully understood. These are balanced by the regulatory activities of T cell and non-T cell populations, both at the early stages of T cell activation in the lymphoid system and within the CNS itself once inflammation is underway.

To better understand the regulation of CNS inflammation we have focused on some key molecules involved in promoting EAE pathogenesis (e.g., IL-6, GM-CSF, T-bet) and others involved in resolution (e.g., IL-10, IFN-γ).

B cells are an important source of IL-10, limiting the size of the encephalitogenic cohort that develops. This effect seems restricted to the lymphoid system. The key IL-10-producing population within the CNS itself are Foxp3+ T regulatory (Treg) cells. Mice that lack either of these IL-10-producing populations fail to recover from EAE.

IL-6 is required to drive pathogenic function in T cells. Although B cells can also provide IL-6, this function is not required. Instead, dendritic cells provide a sufficient and short-lived source, early after T cell activation. T cells later lose IL-6 receptor expression such that those in CNS (both effector and Treg populations) are insensitive to IL-6. This predicts that IL-6-blocking strategies might not influence T cell autoaggression during ongoing disease, but also explains why CNS Treg resist conversion to IL-17-producing effector function. Gene expression studies highlight marked differences between the spleen and CNS in both the T effector and the Treg populations, providing possible mechanistic insights into Treg function and new therapeutic opportunities.

As part of this, the ability of CNS Treg to modulate their expression of cytokine receptors, and therefore their sensitivity to cytokines that might divert them from their regulatory duties, seems a key feature in maintaining Treg function in the face of tissue inflammation.

